# Mesenteric-Portal Vein Resection during Pancreatectomy for Pancreatic Cancer

**DOI:** 10.1155/2015/659730

**Published:** 2015-11-01

**Authors:** Valentina Beltrame, Mario Gruppo, Sergio Pedrazzoli, Stefano Merigliano, Davide Pastorelli, Cosimo Sperti

**Affiliations:** ^1^Department of Surgery, Oncology and Gastroenterology, 3rd Surgical Clinic, University of Padua, 35128 Padua, Italy; ^2^Department of Oncology, Veneto Institute of Oncology, 35128 Padua, Italy

## Abstract

The aim of the present study was to determine the outcome of patients undergoing pancreatic resection with (VR+) or without (VR−) mesenteric-portal vein resection for pancreatic carcinoma. Between January 1998 and December 2012, 241 patients with pancreatic cancer underwent pancreatic resection: in 64 patients, surgery included venous resection for macroscopic invasion of mesenteric-portal vein axis. Morbidity and mortality did not differ between the two groups (VR+: 29% and 3%; VR−: 30% and 4.0%, resp.). Radical resection was achieved in 55/64 (78%) in the VR+ group and in 126/177 (71%) in the VR− group. Vascular invasion was histologically proven in 44 (69%) of the VR+ group. Survival curves were not statistically different between the two groups. Mean and median survival time were 26 and 15 months, respectively, in VR− versus 20 and 14 months, respectively, in VR+ group (*p* = 0.52). In the VR+ group, only histologically proven vascular invasion significantly impacted survival (*p* = 0.02), while, in the VR− group, R0 resection (*p* = 0.001) and tumor's grading (*p* = 0.01) significantly influenced long-term survival. Vascular resection during pancreatectomy can be performed safely, with acceptable morbidity and mortality. Long-term survival was the same, with or without venous resection. Survival was worse for patients with histologically confirmed vascular infiltration.

## 1. Introduction

Involvement of peripancreatic large blood vessels is detected in about half of pancreas cancers [1] and has been generally considered a contraindication to surgery, for many years. In fact, vascular resection was widely considered a difficult surgical procedure with high incidence of complications [2, 3] and without significant effectiveness in survival, compared to palliative operations [4, 5]. Nevertheless, surgical resection is still considered the only curative opportunity for patients affected by pancreatic cancer and, nowadays, it is considered an effective palliative procedure due to improvement of morbidity and mortality rates after surgery [6–9]. Moreover, in the last two decades, an increasing number of venous resections were reported in centers with high volume of pancreatic surgery, while arterial resection still remains an isolated exception [10].

Aim of this study is to present our single center experience of venous resections during pancreatectomy for pancreatic cancer.

## 2. Patients and Methods

Data were obtained from a retrospective analysis of a prospectively collected database of 241 patients who underwent pancreatectomy for pancreatic cancer from January 1998 to December 2012 at our institution. They were divided in two groups: patients who underwent associated venous (portal-mesenteric) resection (VR+) and those who underwent standard resection (VR−). The two groups were compared in terms of demographic features, surgical procedures, tumor pathologic findings, perioperative outcome, and survival. All surgical procedures were performed by the same surgical team. All patients underwent standardized preoperative assessment: routine blood tests and tumor markers CEA and CA 19-9 determination, abdominal ultrasound (US), computed assisted tomography (CT) scan, and, when needed, magnetic resonance imaging or positron emission tomography (PET). CT with angiographic reconstruction was the preferred imaging for tumor's staging. Limited involvement of superior mesenteric-portal axis (less than 2 cm), in absence of extrapancreatic disease and involvement of superior mesenteric artery and/or celiac trunk, was not considered as contraindication to surgery. No patient underwent neoadjuvant therapy.

Surgical technique included standardized lymph node dissection and para-aortic nodes sampling, pylorus-preserving pancreaticoduodenectomy (PD) for tumors of the head of the pancreas, and distal pancreatectomy with splenectomy for tumors of the body/tail. Total pancreatectomy was performed in selected patients, when resection margin of the pancreas was involved by the tumor or when pancreatic anastomosis was judged at high risk of leakage. In VR+ patients, surgical procedure was similar to standard resection in preparation and exposure of pancreas, with a wide Kocher maneuver. Complete evaluation of vascular invasion was determined after transection of the isthmus; in the last years, we performed translateral approach with dissection of uncinate process first and subsequent en bloc excision of pancreas head and infiltrated vein [11]. Vascular resection technique included tangential resection with linear nonstenosing suture when a focal invasion of superior mesenteric vein or portal vein occurred or excision of infiltrated venous tract and end-to-end anastomosis. In selected cases of longer involvement of vein, a vascular graft with internal jugular vein was performed. Curative resection was defined as tumor's resection with pathologically confirmed negative margins. R1 resection was defined as the presence of tumor ≤ 1 mm from the margin, according to Leeds criteria [12]. Perioperative morbidity and mortality were investigated in both groups: operative mortality was defined as death within 30 days from operation or during hospitalization. Pancreatic fistula was defined as the drainage of fluid with an elevated level of amylase and graded according to the International Study Group of Pancreatic Fistula recommendations [13]. All patients underwent regular follow-up that included physical examination, abdominal CT or US, and tumor markers determination every 3 months, for the first 2 years, and every 6 months, thereafter.

Survival curves were constructed with the Kaplan-Meier method and compared by the univariate log-rank test: significance was considered as  *p* < 0.05. Tumor's stage, grading, lymph node status, radicality of resection, venous resection, true venous infiltration, and depth of venous involvement were considered as prognostic factors. Independent prognostic variables were evaluated with a Cox regression hazard model. Statistical analysis was performed using the SPSS statistical software package (version 18.0; SPSS Inc., Chicago, IL, USA).

## 3. Results

Clinicopathologic findings of patients included in the study are detailed in Table 1. During the study period, resection rate increased from 18% to 27% in the last 8 years together with the increasing number of vascular resection. A total of 64 patients (26%) underwent venous resection (27 males and 37 females with mean age of 62.5 yrs; range 37–82). We performed 50 pancreatoduodenectomy (PD) procedures, 10 total pancreatectomy (TP) procedures, and 4 distal pancreatectomy procedures with splenectomy (DP) (Table 1). Tangential resection with linear suture was performed in 13 patients and venous resection with end-to-end anastomosis in 50 patients; a prosthetic graft was used in 1 patient. According to the International Study Group of Pancreatic Surgery (ISGPS) classification, [14], 13 patients underwent type 1, 50 patients type 3, and 1 patient type 4 venous resection. All patients exhibited ductal adenocarcinoma at pathologic examination. Histological examination confirmed vascular invasion in 69% of cases; 55 patients (86%) had radical resection (R0), and 9 subjects (14%) had microscopic neoplastic residue (R1). Tumor involved superficial venous wall (up to tunica adventitia) in 8 cases, the tunica media in 24 cases, and the tunica intima in 12 cases. Postoperative outcome is reported in Table 2. Surgical complications occurred in 21 patients (33%): 7 pancreatic fistulas, 4 abdominal effusions, 3 peritoneal bleeding cases, 3 biliary fistulas, 1 bowel perforation, 1 bleeding of digestive tract, and 1 pleural effusion. Three patients underwent reoperation. Postoperative death occurred in 1 (3%) patient. Morbidity rate, types of complication, and reoperation's rate were not statistically different in the two groups of patients: mortality rate in VR− group was 3% versus 4% in the VR− group (Table 2). Two patients experienced portal vein thrombosis 11 and 13 months after operation, respectively. Both patients had endoscopic evidence of gastric varices without episodes of digestive bleeding. After operation, all patients underwent gemcitabine-based adjuvant therapy, associated with radiotherapy in cases of R1 resection.

**Figure 1:**
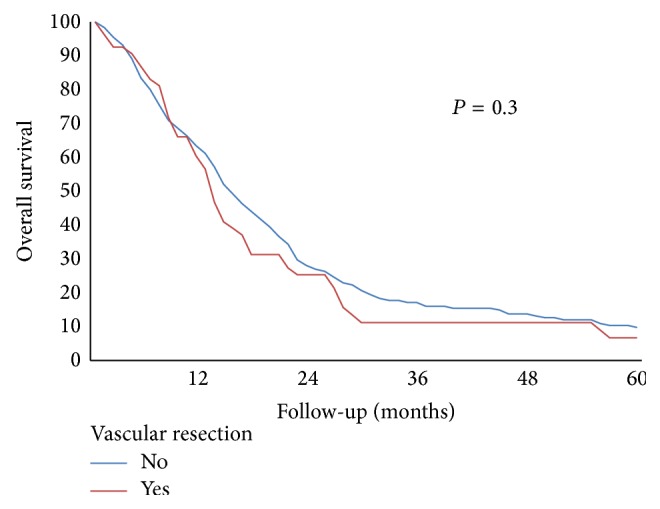
Overall survival in patients who underwent pancreatic resection with or without vascular resection.

**Figure 2:**
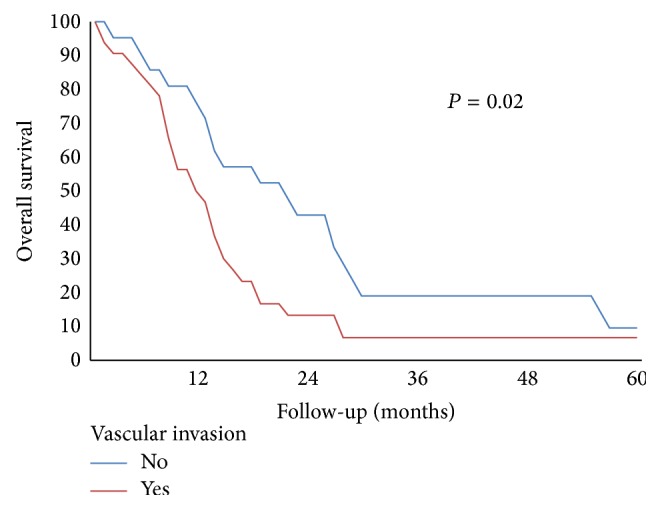
Overall survival in patients who underwent pancreatic resection with vascular resection and histological confirmation of vascular invasion.

Mean and median overall survival time were 24 and 15 months, respectively. Mean survival time was 26 months for patients without vascular resection versus 20 months for VR+ group, while median survival time was 15 and 14 months, respectively (*p* = 0.31) (Table 3). Mean and median survival time for VR+ group without or with histological confirmation of venous involvement were 26 and 22 months versus 17 and 12 months, respectively (*p* = 0.02). Median survival in R0 patients was 17 months versus 10 months in R1 group. Overall survival was 42% at 1 yr, 10% at 2 yrs, and 2% at 3, 4, and 5 yrs (Figure 1). In patients without evidence of vascular invasion, overall survival was 69% at 1 yr, 31% at 2 yrs, 6% at 3 yrs, and 6% at 5 yrs versus 30% at 1 yr and 0% at 2 yrs in subjects with confirmed vascular infiltration (Figure 2). There was a trend of worse prognosis for patients with deeper portal vein invasion without statistical significance (*p* = 0.08).

## 4. Discussion

In our study, we analyzed the outcome after pancreatectomy with or without portal vein resection in a group of 241 patients with pancreatic cancer. Patients with vascular resection had similar outcomes compared to patients who underwent standard resection, with no differences in morbidity, mortality, and long-term survival. Patients had also similar lengths of stay. These results compare favorably with that of other surgical series [15, 16].

The rate of vascular resection during pancreatic operation is strongly influenced by single centers strategies; in some series, associated en bloc vascular resection occurs in few cases [4], and it is depending on careful preoperative selection of patients or intraoperative findings. Other studies present high rates of vascular resections probably due to different behavior related to different persuasions [11]. Our department policy towards resection criteria was to perform venous resection in all cases of small vein involvement (≤2 cm in length), in absence of venous occlusion or thrombosis, in order to obtain an easier end-to-end anastomosis. This approach did not substantially change during the study period, although more operations were performed in the last 6 years. Moreover, we observed a trend of better survival in the last period even though the rate of recurrence after resection was not significantly modified. It is reasonable to believe that this fact could be explained by the introduction of more effective chemotherapeutic regimens (i.e., FOLFIRINOX) for the treatment of relapsing tumors.

Vascular resection and reconstruction could represent a theoretical additional risk in pancreatic-surgical procedures [3]. Nevertheless, morbidity and mortality rates, reported by many centers, are similar to those related to standard surgical technique [15, 16]. In our experience, postoperative complications and mortality rates are comparable with the most recent reports of other institutions, confirming that vascular resection does not increase operative risk. In 2012, Zhou et al. [17] performed a meta-analysis collecting 19 nonrandomized studies, for a total of 2,247 patients. There was no difference in perioperative morbidity and mortality between patients with VR and those without VR. More recently, a meta-analysis of Yu et al. [18] evaluating 22 retrospective studies including 2890 patients confirmed that there was no difference in perioperative morbidity and mortality rates between the two groups of patients, with VR or without VR. However, this study showed differences in median tumor size, R0 resection rate, lymph node metastases, and pancreatic fistula. Although randomized prospective studies are lacking, there are convincing evidences in the literature that pancreatectomy associated with vein resection is safe and feasible and does not increase postoperative morbidity. Thrombosis of the anastomosed vein may occur late after operation, as in our series and in another experience [19] but the risk of digestive bleeding remains only theoretical.

The potential oncological benefit, instead, is still unclear. In our experience, long-term survival was not statistically different between patients who underwent venous resection and those who did not.

Many series of pancreatectomies associated with venous resection show no difference in terms of survival compared to standard resections [20], while other centers report poorer outcome [21]. These differences could be explained by the possibility to obtain a R0 resection; this parameter is known to affect prognosis and is reported with a wide range in other studies (32–87%) [4, 5, 20–22]. In our experience, R0 resections rate was 86% with a median survival of 17 months versus 10 months for R1 patients. Furthermore, only patients with R0 resection were alive for 2 years, even in absence of statistical significance. Recently, in a prospective multicentric study, Delpero et al. [23] reported an increasing frequency of positive resection margins in patients who required portal-mesenteric vein resection: this finding was previously showed by other authors [24] and associated with tumor's size and biology, rather than with vascular resection.

Regarding histological confirmation of vascular infiltration, this parameter was not confirmed at histological analysis in 31% of our cases, with a median survival of 16.5 months versus 9.5 months in patients with histological report of vascular invasion (*p* = 0.02). These data suggest a satisfactory oncological benefit of vascular resection especially in selected patients with inflammatory vascular adherence [25]. However, better survival after pancreatectomy with venous resection compared to palliative surgical bypass has been reported [16]. Moreover, it is difficult to distinguish true neoplastic involvement from inflammatory adhesion both pre- and intraoperatively, so whenever possible, pancreatectomy with vascular resection is advisable to reach a radical resection together with the best tumor's staging. The depth of tumor invasion has been shown to be a negative prognostic factor. Fukuda et al. [26] showed that the deeper invasion (tunica media or intima) was an independent prognostic marker for poorer survival, with median survival similar to that of nonresected patients. In our experience, there was a trend to worse prognosis when deeper portal vein wall invasion occurred, but without statistical significance. Further studies are necessary to evaluate the depth of vein involvement as a possible contraindication to resection.

## 5. Conclusion

In conclusion, vascular resection seems to offer an oncological benefit when it increases tumor's resectability and R0 resection rates [27], in absence of other parameters affecting prognosis. Vascular resection presents acceptable perioperative morbidity and mortality rates, when performed in specialized centers; nevertheless, long-term survival rates are still conflicting and careful evaluation and selection of patients are recommended.

## Figures and Tables

**Table 1:** Clinicopathological features of patients subjected to pancreatic resection with (VR+) and without (VR−) vascular resection.

	VR+	VR−
	(*n* = 64)	(*n* = 177)
Age		
Median	60.7	63.7
Range	(37–82)	(37–84)
Gender		
M	23	95
F	30	82
pT1	0	3
T2	3	21
T3	22	149
T4	28	4
Lymph node		
Yes	39	117
No	14	60
Grading		
G1	14	29
G2	28	95
G3	11	53
Radicality		
R0	55	125
R1	9	46
R2	0	6
Type of resection		
PD	50	121
TP	10	6
DP	4	50

PD: pancreatoduodenectomy; TP: total pancreatectomy; DP: distal pancreatectomy.

**Table 2:** Postoperative outcome of patients after pancreatic resection with (VR+) and without (VR−) vascular resection.

	VR+	VR−
	(*n* = 64)	(*n* = 177)
	*n* (%)	*n* (%)
Complications		
** **Pancreatic fistula	7 (11)	23 (13)
** **Biliary fistula	3 (5)	5 (3)
** **Fluid collections	4 (6)	9 (5)
** **Peritoneal bleeding	3 (5)	9 (5)
** **Digestive bleeding	1 (2)	1 (0.5)
** **Ileal perforation	1 (2)	5 (3)
** **Pleural effusion	1 (2)	2 (1)
** **Others	1 (2)	6 (3)
Reoperation	**4 (** **6)**	**9 (** **5)**
Morbidity	**19 (29%)**	**53 (30%)**
Mortality	**2 (3%)**	**7 (4%) **

PD: pancreatoduodenectomy; TP: total pancreatectomy; DP: distal pancreatectomy.

**Table 3:** Univariate analysis of factors influencing overall survival.

Variables	Survival (mo)		
	Mean	Median	Significance
Grading			
Well-moderate	26.7	17	*p* = 0.009
Poor	19.8	14	
Node status			
No	24.7	16	*p* = 0.977
Yes	23.6	15	
Radicality			
No	26.6	17	*p* = 0.001
Yes	19.5	10	
Vein resection			
No	25.8	15	*p* = 0.301
Yes	19.9	14	
